# Association of long-term consumption of repeatedly heated mix vegetable oils in different doses and hepatic toxicity through fat accumulation

**DOI:** 10.1186/s12944-020-01256-0

**Published:** 2020-04-13

**Authors:** Gul Ambreen, Afshan Siddiq, Kashif Hussain

**Affiliations:** 1 Department of Pharmacy, Aga Khan University Hospital, Stadium Road (Main Pharmacy), P.O Box 3500, Karachi, 74800 Pakistan; 2 Department of Pharmacology, University of Karachi, Karachi, Pakistan

**Keywords:** Hepatic toxicity, Oxidative stress, RHMVO, STHMVO, Repeatedly heated mix vegetable oils

## Abstract

**Background:**

Hepatic diseases are one of the chief reasons for worldwide morbidity and mortality. The increased incidence in Asian countries is driving researchers to explore preventive ways from nature. It is more practical to go with healthy routine edibles like vegetable oils to avoid environmental and chemical hepatic injuries. With the use of thermally oxidized oils overproduction of reactive oxygen species (ROS) with overwhelmed cellular antioxidants defense system results in oxidative stress, the known cause of cardiovascular diseases (CVDs), cancers and neurodegenerative disorders. Little is investigated about the effect of daily used oxidized cooking oils on hepatic function changes with oxidative stress especially in the animal model that mimics the human situation.

**Methods:**

In this study, healthy adult male rabbits of local strain were divided into 4 groups (*n* = 12). First, two sets of rabbits were treated with 1 and 2 ml/kg/day of repeatedly heated mix vegetable oils (RHMVO) respectively. The third set of rabbits was given 1 ml/kg/day of single time heated mix vegetable oils (STHMVO) and the fourth set of rabbits served as controls and fed with normal rabbit diet to for 16 weeks. Serum liver function markers including total-protein, albumin, serum glutamic-oxaloacetic transaminase (SGOT), serum glutamic-pyruvic transaminase (SGPT) and alkaline phosphatase (ALP) along with the activity of hepatic antioxidant-enzymes including superoxide dismutase (SOD), catalase (CAT), glutathione peroxidase (GPx) and malondialdehyde (MDA) for lipid peroxidation were compared among different groups of rabbits. Histopathological examination was performed for all four groups.

**Results:**

Significantly (*p* < 0.05) elevated hepatic enzymes and MDA levels, with lower total protein, serum albumin, GPx, SOD and CAT levels were found in high and low doses RHMVO treated groups, in comparison to control. In the STHMVO group, all mentioned markers were insignificantly changed. Accumulation of liver fat in low and high dose oil-treated groups was further confirmed under the microscopic examination of liver tissues, presented significant fat accumulation in liver tissues, in addition, 40–60% increased oxidative stress compared to control, in a dose-dependent manner.

**Conclusions:**

These results conclude that consumption of thermally oxidized mix vegetable oils for longer duration can impair the liver function and destroy its histological structure significantly through fat accumulation and oxidative stress both in high as well as low doses.

## Background

Vegetable oils are an essential part of the human diet, being the major lipid and energy source, which also serve as an integral part of bio-membrane and hormonal building block [1] Cooking oils are mostly consumed in food processing to enhance palatability of the food. In households and at the commercial level, to minimize the cost and maximize profits, cooking oil is often reused, which is common practice in South Asian countries like Pakistan. Several studies have reported the harmful effect of thermal oxidation on cooking oils and processed food [2], but regardless of all these studies, the practice seems to continue.

Physico-chemical characteristics of the cooking oils changes when heated beyond a certain limit and several chemical reactions take place in moisture and air presence, oils degenerate and produce volatile substances, unwanted monomers, polymers, isomers and free radical [3]. In cooking oil fatty acids (FAs) naturally exist in the cis-isomer form, but during thermal oxidation they convert into trans-isomers, possessing physical properties similar to saturated FAs [2]. Several animal studies demonstrate that consumption of repeatedly heated oils increased the risk of cardiovascular diseases (CVDs) like hypertension with reduced vasorelaxation responses [4], endothelial malfunction [5], lipid peroxidation [6], atherosclerosis [7], oxidative stress [8], genotoxicity [9], carcinogenicity and lower glucose absorption [10].

Trans-isomer FAs consumption has been associated with the occurrence of metabolic syndrome (obesity, CVDs, insulin resistance along with systemic inflammation) [11]. Systemic inflammation induced imbalance of prostaglandins causes thrombogenesis [12] and activation of systemic inflammatory responses [13]. Oxidative stress and damage is a chief contributing factor to the development of CVDs [14]. About three-quarters of all deaths due to CVDs were only reported from countries with low to middle income [15]. The South Asian population has the highest known rate of coronary artery diseases with the predicted addition of up to about twenty-four million by 2035 [16]. The liver, colorectal & stomach cancers are among the top five frequently detected reasons for deaths due to cancer [17].

The liver is the vital part of our body which is responsible for detoxification of toxic metabolites, protein synthesis and productions of compounds required for food digestion [18]. Excessive free radicals are known to cause liver damage and may result in hepatitis, cirrhosis and hepatic tumor [19]. Thermally oxidized oils are the most significant source of oxidative damage in our body if used regularly. Hepatic problems are among the leading reasons for worldwide morbidity and mortality [20]. A highly increased number of hepatic diseases in Asian countries [21, 22] is also driving researchers to explore the natural therapeutic and protective agents for hepatic problems [23]. However, little is investigated about the effect of daily used oxidized cooking oils on changes in hepatic functions with induced-oxidative-stress [24] especially in the model that mimics the human situation.

Overproduction of reactive oxygen species (ROS), like hydrogen peroxide (H_2_O_2_), superoxides and hydroxyl, with overwhelming cellular antioxidants defense system results in oxidative stress [25–27], the known cause of CVDs, cancers and neurodegenerative disorders [28]. ROS are removed from the biological system through a number of enzymes and non-enzymatic defense mechanisms [29]. In enzymatic eradication, superoxide anions are converted to H_2_O_2_, and further rapidly degrade H_2_O_2_ to H_2_O by first line antioxidants defense system of superoxide dismutase (SOD), catalase (CAT), and glutathione peroxidase (GPx) [29, 30]. Furthermore, oxidative stress-induced uncontrolled lipid peroxidation may also result in cell injury through protein inhibition and DNA damage [31, 32]. Malondialdehyde (MDA), a stable end product of lipid peroxidation, is used for measuring the collective lipid peroxidation. Thus, based on the previous studies, SOD, CAT and GPx, MDA have been studied as biological markers of oxidative stress and lipid peroxidation [30, 33].

Presently, several animal models have been used to study fat-induced pathological conditions, like mice, zebrafish and, rats. To study the effect of differently treated vegetable oils on lipid metabolism and oxidative stress, the selection of experimental animal models is very critical to conduct basic research and develop a study tool. In this respect, to study human fat metabolism the rabbit has become the most appropriate animal model because of rabbits’ unique features of lipid metabolism similar to humans [34–36].

Previous animal studies have reported that 57% caloric requirements from the fat diet resulted in hepatic steatosis, damage and non-alcoholic fatty liver disease [37–39]. In all these studies a single oil was investigated not the blend of thermally oxidized vegetable oils. In Pakistan commercially available oils are mostly a blend of two or more edible oils and the most common available blend of an equal ratio of olive, canola and sunflower oils was focused in this study, to have the model that mimics the human situation. To our information, no such effort has been reported from Karachi, Pakistan, in particular. We designed this animal model to investigate the development of hepatic toxicity with induced oxidative-stress through the long-term consumption of repeatedly heated mix vegetable oils (RHMVO) in high and low doses in comparison with single time heated mixed vegetable oils (STHMVO) and control, in rabbits [34].

## Methods

### Oil samples preparation

Standard food-grade canola, sunflower and olive oils (7 l of each) were purchased from the Karachi local market. Each oil was thermally treated for a total of 45 min till 330 to 360 °F (beyond the smoke point) [40, 41], then cooled to room temperature and mixed in equal ratio (1:1:1) then sufficient quantity of oil labeled as STHMVO. The remaining oil mixture was heated in the same manner for the coming 9 days (one time each day) and then cooled to room temperature. Every day before heating, the oil level was adjusted in the pan with the same oil blend (0.007 ± 0.003 l per day) to meet the initial level. This replenishment method was adopted to replicate the same practices used in fried and fast food outlets [40]. At the end of the 10th day, viscous dark brown oil was obtained. To prevent photodegradation it was stored in amber color bottles and labeled as RHMVO.

### Experimental animals

Healthy adult male rabbits of local strain weighing between 1450 ± 20 g were purchased from the animal house of Department of Pharmacology, University of Karachi, Pakistan. Rabbits were kept individually in wire-topped steel cages with a wooden bottom, provided control conditions of relative humidity (50–60%) and temperature (23 ± 2 °C) for 16 weeks with an estimated 12/12 h light/dark photo-cycle. Animals were acclimatized with the above-mentioned conditions for seven days before starting the experiments. Animals were handled according to the institutional animals’ ethical committee guidelines.

### Experimental procedure

Doses were calculated based on grams per kg [42] and higher doses (double the normal) of oil were used to mimic the high intake with fried food. The daily recommended amount of edible vegetable oils for human adult males is 35–55 g per day (0.94 g/ml) [43]. The average body mass of an Asian male adult is around 57.7 kg [44]. On the basis of these facts normal daily dose of the oil is calculated to be 1 ml/kg/day. To imitate the higher intake of the oil with fried food, the dose of 2 ml/kg/day was calculated. Oil consumption, duration of 16 weeks was selected (equivalent to 12 years of human life). The study was performed by randomly allocating rabbits in the following treatment categories and the control group (*n* = 12).

- Control group: Unexposed control rabbits
- STHMVO group: animals were orally administered STHMVO (1 ml/kg/day)
- L-RHMVO group: rabbits were orally administered RHMVO (1 ml/kg/day)
- H-RHMVO group: rabbits were orally administered RHMVO (2 ml/kg/day)

Animals of all the groups had free access to water. The animals of study groups were administered respective oils in predefined doses, daily at 10 am through the oral route with the help of a syringe. For the rest of the day animals of all the groups were fed ad libitum on a regular diet (fresh hay and water). Control group animals were fed on fresh hay and water only. Morbidity and mortality were monitored. Animal’s body weight was recorded at baseline followed by weekly monitoring for 4 weeks, then on weeks 8, 12 and 16. At the completion of the study period (16 weeks) animals were euthanized through the intravenous administration of sodium pentobarbital 100 mg per kg followed by decapitation [45]. Furthermore, animals were necropsied for histopathological examination of internal organs and to retrieve the liver for organ weight evaluation. Food and water intake were recorded for each group.

### Blood sample collection and liver function markers analysis

Blood was collected from the ear vein of animals in a fasting state after the completion of 16^th^week. For hematological parameter study, 5 ml blood was collected in gel tubes for biochemical analysis. Blood was centrifuged at 3000 rpm for 15 min duration to isolate the serum. With the use of clean, dry disposable plastic syringes the serum was collected and stored at a temperature of − 20 °C and then used to analyze for total-protein, albumin, serum glutamic-pyruvic transaminase (SGPT), serum glutamic-oxaloacetic transaminase (SGOT) and alkaline phosphatase (ALP) using analytical kits of RANDOX Laboratories Ltd. following the manufacturer’s instructions.

### Lipid peroxidation- malondialdehyde measurement

Lipid-peroxidation in tissue homogenate was evaluated as per the Wills method (1969). Absorbance was measured at 532 nm. Malondialdehyde (MDA) amount is expressed in nano-moles/gram of tissue wet-weight.

### Hepatic antioxidant-enzymes activity measurement

To assay liver, homogenate activity of superoxide dismutase (SOD) Beyer and Fridovich method [46] was followed using spectrophotometric analysis. Inhibition of nitro blue tetrazolium (NBT) oxidation by SOD was measured. A unit of superoxide dismutase represented the SOD requirement to inhibit a 50% rate of NBT oxidation at 25 °C. Activity is shown in the unit per mg of protein. With slight changes in Euler’s method [47], the activity of catalase (CAT) was measured. The capacity of CAT to decompose hydrogen peroxide was measured at 240 nm. Used extinction-coefficient of 0.043 mM-1 cm-1. One unit of activity is defined as hydrogen peroxide degraded /minute/mg protein. Flohe and Gunzler method was followed for measurement of activity of glutathione peroxidase (GPx), articulated at 25 °C in mmol of GSH oxidized/minute per mg of protein.

### Microscopic examination

After getting the weight of the liver, the tissue samples were treated with alcohol to dehydrate. After cleaning with xylene, samples were further embedded in paraffin at 56 °C, (melting point). By using rotatory microtome 5 μm sized sections were obtained. After flattening on warm water, they were mounted onto albumerised slides and keep for drying for 12 h. Dewaxing in xylene and hydration with ethanol and water was performed. The sections were first treated with Harris haematoxylin to stain, thereafter differentiation by acid alcohol then staining in methylene blue was performed. Furthermore, dehydration of sections in alcohol (95%) and staining in alcohol eosin (10%), dehydration with absolute alcohol and cleaning with xylene were completed. The sections were then mounted using Canada balsam. These prepared slides were finally viewed with the use of a light microscope.

### Statistical analysis

In our study, the final data were accessible in the form of mean ± standard deviation, 95% CI and CV%. We applied analysis of variance (ANOVA) followed by post hoc Tukey’s Honest Significant Difference (HSD) test to find out statistical significance at *p* < 0.05. Statistical Product and Service Solutions software was used.

## Results

### Nutritional status and growth

**Fig. 1:**
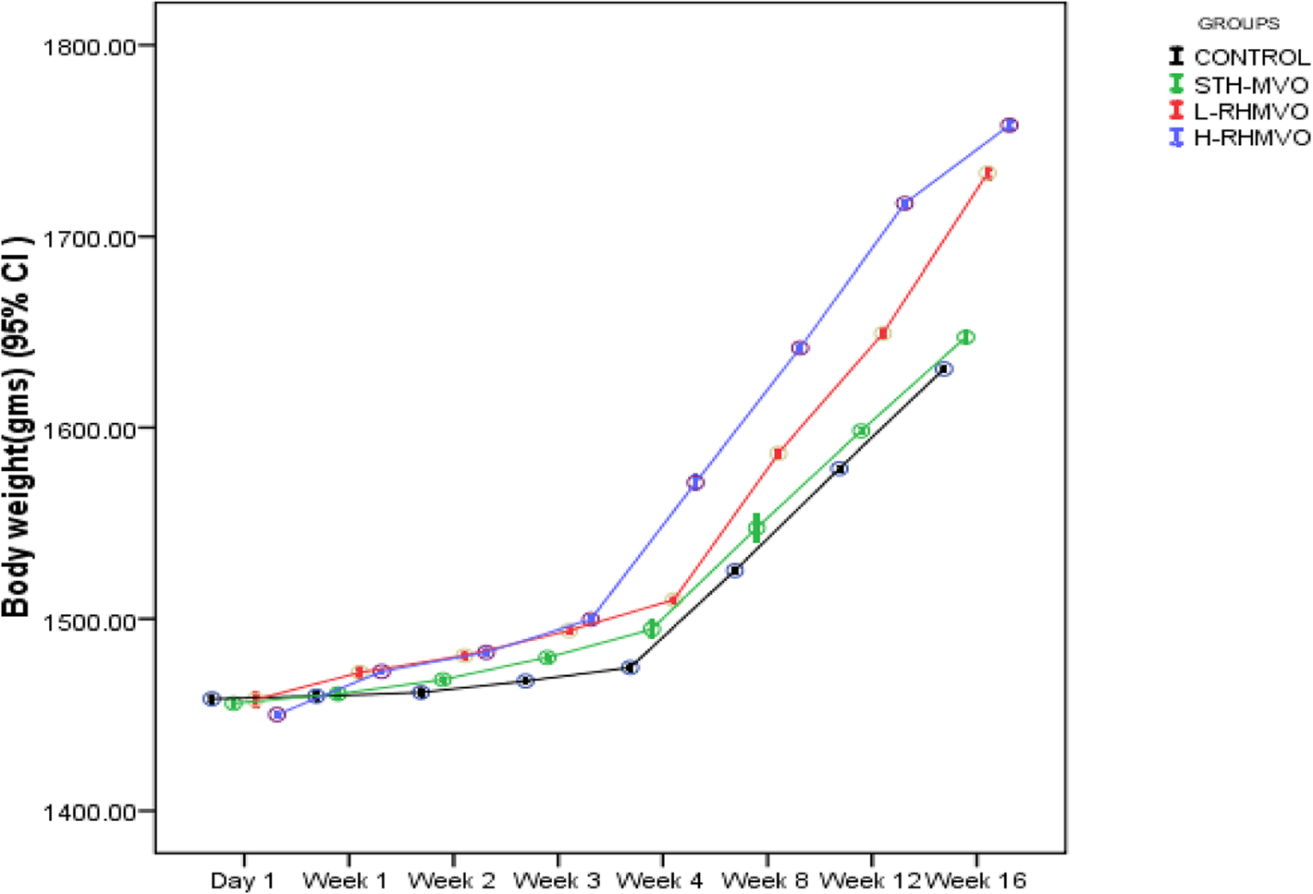
Comparison of weight gain in rabbits from the control and different oils (STHMVO, L-RHMVO & H-RHMVO) fed groups at 16 weeks (n = 12)

Weight changes (body and liver) were observed in all the groups consistently for 16 weeks. As shown in (Table 1) statistically significant weight gain was observed at both high and low doses treated RHMVO despite lowered food and water intake (Fig. 1). In STHMVO treated group body and liver weight gain along with food and water intake was insignificantly different from control.

**Table 1:** Comparison of nutritional status and growth in rabbits from the control and different oils (STHMVO, L-RHMVO & H-RHMVO) fed groups at 16 weeks (*n* = 12)

**Groups**	**BW gain (gms)**			**Food intake (gms /day)**			**Feed efficiency**
	**Mean** ± **SD**	**95% CI**	**CV%**	**Mean** ± **SD**	**95% CI**	**CV%**	**Ratio (BW gain/food intake)**
Control	177.9 ± 0.5	175.2 to 180.1	0.3	122.4 ± 3.9	119.9 to 124.8	3.2	1.45
STHMVO	189.4 ± 0.8	186.0 to 192.2	0.4	120.8 ± 3.5	118.6 to 123.1	2.9	2.17
L-RHMVO	258.7 ± 1.3*	252.5 to 259.9	0.5	98.2 ± 3.2*	96.2 to 100.2	3.3	2.63
H-RHMVO	259.9 ± 1·5*	252.1 to 261.4	0.6	95.5 ± 2.9*	93.6 to 97.4	3.0	2.72
	**Liver weight (gms)**			**Water intake (ml/day)**			**Liver weight (% of body weight)**
	**Mean** ± **SD**	**95% CI**	**CV%**	**Mean** ± **SD**	**95% CI**	**CV%**	(Liver wt/BW)* 100
Control	55.8 ± 0.4	54.7 to 56.9	0.7	117.1 ± 3.5	114.9 to 119.4	3.0	31.3
STHMVO	58.5 ± 0.5	57.3 to 59.8	0.9	124.5 ± 2.90*^a^	122.6 to 126.3	2.3	30.8
L-RHMVO	82.9 ± 0.4*	82.0 to 83.9	0.5	97.7 ± 2.0*	96.4 to 98.9	2.0	32.1
H-RHMVO	87.4 ± 1.5*	83.7 to 91.2	1.7	88.1 ± 1.6*	87.2 to 89.1	1.8	33.6

* The mean difference is significant at *p* < 0.05. *a water intake significantly more than the control group. *BW* body weight, *SD* standard deviation, *CI* confidence interval, *CV* coefficient of variation, *STHMVO* single time heated multiple vegetable oil, *L-RHMVO* low dose- repeated heated mix vegetable oil, *H-RHMVO* high dose- repeated heated mix vegetable oil

### Biochemical markers of hepatic-function

Plasma level of hepatic-function markers; serum glutamic-pyruvic transaminase (SGPT), serum glutamic-oxaloacetic transaminase (SGOT), alkaline phosphatase (ALP) was increased, and level of albumin and total protein were significantly decreased (*p* < 0.05) in both low and high RHMVO fed groups in comparison with control and STHMVO fed group (Table 2). Insignificant changes were observed in the STHMVO group in comparison with control.

**Table 2:** Comparison of plasma liver-specific enzymes in rabbits from the control and different oils (STHMVO, L-RHMVO & H-RHMVO) fed groups at 16 weeks (*n* = 12)

Groups	Mean ± SD	95% CI	CV%
**SGPT(U/L)**			
Control	88.8 ± 7.3	84.2 to 93.5	8.2
STHMVO	94.7 ± 6.8	90.4 to 99.0	7.2
L-RHMVO	195.6 ± 9.2 *	189.7 to 201.4	4.7
H-RHMVO	245.7 ± 4.1 *	243.2 to 248.3	1.7
**SGOT(U/L)**			
Control	86.2 ± 8.3	80.8 to 91.4	9.6
STHMVO	92.1 ± 6.1	88.2 to 95.9	6.6
L-RHMVO	194.8 ± 4.6 *	191.9 to 197.7	2.4
H-RHMVO	255.8 ± 7.2 *	251.2 to 260.3	2.8
**ALP(U/L)**			
Control	79.7 ± 8.5	64.6 to 83.7	10.7
STHMVO	98.2 ± 5.6	94.6 to 101.7	5.7
L-RHMVO	141.4 ± 6.7 *	137.1 to 145.6	4.7
H-RHMVO	227.8 ± 3.4 *	225.6 to 229.9	1.5
**Toal Protein (mg/dl)**			
Control	7.9 ± 0.8	7.4 to 8.4	10.1
STHMVO	7.5 ± 0.8	7.5 to 8.1	10.6
L-RHMVO	3.9 ± 0.3 *	3.7 to 4.1	7.7
H-RHMVO	3.6 ± 0.5 *	3.4 to 4.0	13.8
**Albumin (mg/dl)**			
Control	3.4 ± 0.7	2.9 to 3.8	20.5
STHWMVO	3.2 ± 0.4	2.9 to 3.4	12.5
L-RHMVO	1.7 ± 0.4 *	1.4 to 1.9	23.5
H-RHMVO	1.4 ± 0.1 *	1.3 to 1.4	7.1

* The mean difference is significant at *p* < 0.05.; *SD* = standard deviation; *CI* confidence interval, *CV* coefficient of variation, *STHMVO* single time heated multiple vegetable oil, *L-RHMVO* low dose- repeated heated mix vegetable oil, *H-RHMVO* high dose- repeated heated mix vegetable oil, *SGPT* serum glutamic-pyruvic transaminase, *SGOT* serum glutamic-oxaloacetic transaminase, *ALP* alkaline phosphatase

### Antioxidant-enzymes and malondialdehyde level

**Fig. 2:**
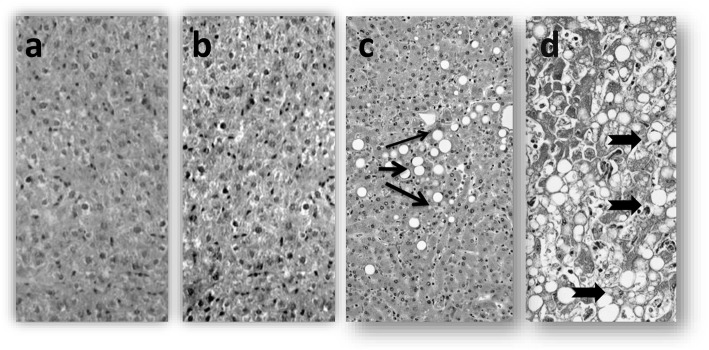
Histopathological images of rabbits liver after 16-week treatment: Control (**a**), Normal rabbit liver, STHMVO treated (**b**) observed an insignificant number of fibrous tissues in the portal area. L-RHMVO treated (**c**) fibrosis found around the portal area with fibrous septa forming in the lobule and H-RHMVO treated (**d**) a large number of fibrous septa formed. Which damaged the hepatic lobule (× 200)

Antioxidant enzymes like catalase (CAT), glutathione peroxidase (GPx), and superoxide dismutase (SOD) were significantly (*P* < 0.05) decreased in L-RHMVO and H-RHMVO groups in a dose-dependent manner in comparison with STHMVO and control (Table 3). In the analysis of lipid peroxidation in the organs showing lipidosis, along with significantly elevated malondialdehyde (MDA) levels in L-RHMVO and H-RHMVO groups. In microscopic findings of the liver, extensive lipid accumulation was observed in L-RHMVO and H-RHMVO group rabbits (Fig. 2).

**Table 3:** Comparison of hepatic-antioxidant enzyme and malondialdehyde levels in rabbits from the control and different oils (STHMVO, L-RHMVO & H-RHMVO) fed groups at 16 weeks (n = 12)

Groups	Mean ± SD	95% CI	CV%
**CAT (U/mg)**			
Control	207.2 ± 22.5	196.4 to 219.3	10.9
STHMVO	206.8 ± 19.3	194.5 to 219.0	9.3
L-RHMVO	148.9 ± 15.5 *	139.1 to 158.7	10.4
H-RHMVO	140.5 ± 12.4 **	132.6 to 148.4	8.8
**SOD (U/mg)**			
Control	29.3 ± 1.5	28.4 to 30.2	4.9
STHMVO	29.9 ± 1.4	28.0 to 30.9	4.6
L-RHMVO	13.3 ± 1.3 *	12.5 to 14.1	9.5
H-RHMVO	7.7 ± 0.6 *	7.3 to 8.1	7.2
**GPx (U/mg)**			
Control	34.6 ± 1.4	33.8 to 35.5	3.9
STHMVO	33.8 ± 1.3	33.0 to 34.6	3.7
L-RHMVO	19.6 ± 1.5 *	18.7 to 20.5	7.4
H-RHMVO	8.3 ± 0.5 *	7.9 to 8.6	6.1
**MDA (nmol/g)**			
Control	15.5 ± 1.6	14.4 to 16.5	10.5
STHMVO	15.0 ± 1.4	14.2 to 15.9	9.1
L-RHMVO	39.5 ± 1·9 *	38.3 to 40.6	4.7
H-RHMVO	42.5 ± 5.2 **	39.2 to 45.8	12.2

* The mean difference is significant at *p* < 0.05. ** The mean difference is highly significant at *p* < 0.005. *SD* standard deviation, *CI* confidence interval, *CV* coefficient of variation, *STHMVO* single time heated multiple vegetable oil; L-*RHMVO* low dose- repeated heated mix vegetable oil, *H-RHMVO* high dose- repeated heated mix vegetable oil, *SOD* superoxide dismutase, *CAT* catalase, *GPx* glutathione peroxidase, *MDA* malondialdehyde

## Discussion

Following long term oil treatment, the body and liver weight gain in RHMVO fed animals, both in high and low doses was statistically significant (*p* < 0.05) in comparison with control. In addition, the feeding-efficiency of RHMVO treated animals was lesser than STHMVO fed rabbits. That may be clarified as nearly all amino acids (AAs) react with oxidized lipids and their primary and secondary product, thus leading to decreased digestive utilization of AAs, proteins and fats, a contributing factor for weight gain [48]. The weight variances among the oil feed groups are in line with Sayon-Orea et al. study, which reported that fried edible products increased central adiposity and blood pressure [49]. Other evidence of increased risk of obesity, reduced HDL-cholesterol and bigger circumference of waist have been associated with the intake of food products, processed in repeatedly heated oil [49, 50]. The results of our study, with increased body and organ weight but comparatively reduced food intake in RHMVO fed animals are supported by other studies which reported the relationship between indigestibility and repeatedly heated oil consumption [51–53]. This increased body weight might be possibly linked with the deprived pancreatic lipase effect on triacylglycerol, polymers, dimers, and oligomers, present in thermally degenerated oils, as reported in a recent review [54].

The number of biochemical variables of biological-membranes and plasma is strongly influenced by dietary fat. Vegetable oils used in daily life exert their health effect through hepatic protection or damage, depending on factors like quantity used, FAs composition, heating duration, and extent [55, 56]. The results of the current study have shown that repeatedly heated oil-fed groups reported the maximum hepatic damage in a dose-dependent manner, even the intake of low doses may lead to liver toxicity. The results of our study are consistent with previous studies which reported significantly increased serum liver enzyme levels (including SGPT, SGOT, and ALP), which is clearly indicative of hepatic injury [57, 58]. The liver is clearly damaged by excessive free radical and may result in hepatitis, cirrhosis and hepatic tumor [19]. Thermally oxidized oil is the most significant source of oxidative damage for human health if used daily for a long time. In H-RHMVO groups, highly elevated levels of SGOT (sensitive and a precise biomarker for hepatotoxicity) and ALP (the main biomarker of hepatic and biliary defects like cholestasis) [59] reflected hepatocytes damage and tissue interruption, allowing the leakage of intracellular enzymes in the blood [55]. Consistent with the diminished serum albumin and total protein levels and supported by the significantly enlarged liver and histopathological changes in hepatocytes of RHMVO fed rabbits.

The results of hepatic-antioxidant enzyme levels suggest a strong indication of liver lipid-peroxidation with RHMVO intake both in high and low doses. The levels of MDA gauge the lipid peroxidation in the degradative phase. The MDA levels in H-RHMVO and L-RHMVO groups increased very significantly (*p* < 0.005) in comparison with the control group. Although the magnitude of the MDA level was dose-dependent, statistically not significantly different between the low and high dose RHMVO fed groups. Showing the deleterious effect of long-term RHMVO consumption, even in low doses. The finding of our study also confirms that with the consumption of single time heated mix vegetable oils, hepatic lipid-peroxidation was insignificant in comparison with control. Our study confirms that feeding on mixed oxidized oils provides a straight source of oxidative stress for the biological system. The composition of FAs is an important factor as biological membrane adapts, changes as per FAs provided in dietary fats [29, 60, 61], subsequently influence the cellular susceptibility to oxidative stress [57]. With the removal of OH• from a -CH_2_- group of polyunsaturated fatty acids, lipid peroxidation begins. Further conjugated dienes (CDs) are formed by molecular rearrangement. CDs contain 2 double bonds with a single bond as a separator. CDs react with oxygen to form peroxyl radicals, which further react with OH• atom from lipids and produce cyclic peroxides, lipid hydro-peroxides and MDA [29]. Lipid peroxidation in the biological membrane leads to impairment in the membrane functioning, structural integrity and reduced membrane fluidity with compromised enzyme activity present in the membrane [57]. The phospholipids of biological membrane holding trans-fatty acids attract cholesterol [58], leading to structural changes and functional alteration in the signaling mechanism, which further promotes the ROS production [62]. This phenomenon justifies the increased oxidative stress in RHMVO fed animals.

Comparative oxidative stress among all the groups was analyzed by antioxidant enzyme levels. Results show that significant (*p* < 0.05) reduction in SOD, GPx and lower CAT levels in H-RHMVO and L-RHMVO groups in comparison with control. Other South Asian region animal studies also support our results, but all these studies were conducted with single edible oil [42]. These results suggest that consumption of thermally oxidized oils may cause genotoxicity and carcinogenicity [63, 64]. As altered antioxidant activity further leads to oxygen intolerance, protein and DNA oxidation and finally cellular death [29]. In addition, antioxidant enzyme activity is inhibited by the toxic intermediates formed due to high-fat diet consumption [65] and accumulated H_2_O_2_ and oxygen-radicals also support the rapid formation of hydroxyl radicals [66]. This study emphasizes that the consumption of thermally oxidized mix vegetable oils is associated with a significantly reduced hepatic antioxidant-enzymatic system and induced hepatic-oxidative stress and pro-inflammatory state in rabbits. Further advance animal and human studies are required to evaluate the formation of DNA adducts with thermally oxidized oils in a model that mimics the human situation, considering the food processed in oxidized oils and roadside contamination being the part of the study design.

In normal routine consumers get exposed to several complex mixtures of substances and environmental chemicals so one-exposure-one-health-effect is not a realistic approach [67] but in a developing country like Pakistan, this study will provide information on real-life ground realities to divert attention towards these unaddressed issues. This study will also help to shift the paradigm to select the study models that mimic the human situation in developing societies.

## Conclusion

In conclusion, this study highlighted that long-term consumption of oxidized mix edible oils induced hepatic-toxicity and lipid-peroxidation with induced oxidative stress in comparison with single time heated mixed vegetables oils. As the blend of oils was the same in single and multiple times heated oils mixture, so we conclude that trans-isomerization of fatty acids during thermal oxidation has the strongest association with hepatic oxidative stress rather than only the composition of fatty acids in the oil blend. This study also concludes that the consumption of thermally oxidized oils blend even in lower amounts is deleterious to health. This study provides strong evidence of hepatic dysfunction for lipid metabolism with a daily intake of low and high doses of thermally oxidized mixed vegetable oils, a key factor of the cardio-metabolic syndrome. This study highlights the knowledge, awareness of harmful health effects of eating food processed in RHMVO and also highlights the importance of conducting further animal and human studies in a model that mimics the real-life scenario in developing countries, to evaluate the reasons for increased incidences of CVDs and cancer in the South Asian region.

## Data Availability

All data generated or analyzed during this study are included in this published article. The datasets used and/or analyzed during the current study are available from the corresponding author on reasonable request.

## Abbreviations

ROS: Reactive oxygen species
SGOT: Serum glutamic-oxaloacetic transaminase
SGPT: Serum glutamic pyruvic transaminase
ALP: Alkaline phosphatase
SOD: Superoxide dismutase
CAT: Catalase
GPx: Glutathione peroxidase
MDA: Malondialdehyde
CDs: Conjugated dienes
